# Effects of Reduced-Level Coated Inorganic Compound Trace Minerals on Breeder Hen Performance, Eggshell Quality, Antioxidant Status, and Progeny Development Compared with an Inorganic Premix

**DOI:** 10.3390/vetsci13080842

**Published:** 2026-08-21

**Authors:** Linfeng Zhou, Zhongyu Li, Ruicheng Han, Lubing Xu, Hao Sun, Liangmei Xu, Hongzhi Wu

**Affiliations:** 1 College of Animal Science and Technology, Northeast Agricultural University, Harbin 150030, China; linzhou152@163.com (L.Z.); potato_li@163.com (Z.L.); hrc20030514@163.com (R.H.); bingxu0120@163.com (L.X.);; 2 Tropical Crop Genetic Resource Research Institute, Chinese Academy of Tropical Agricultural Sciences, Haikou 571101, China

**Keywords:** coated inorganic compound trace elements, egg breeder hen, egg quality, eggshell ultrastructure, intestinal development

## Abstract

This study evaluated the effects of reduced inclusion levels of coated inorganic compound trace elements as a reduced substitution for uncoated inorganic trace elements in layer breeder diets. A total of 360 Hy-Line Brown layer breeder hens were randomly assigned to three groups, with six replicate pens of 20 hens per group: control group A (inorganic trace elements), group B (500 mg/kg coated inorganic compound trace elements), and group C (1000 mg/kg coated inorganic compound trace elements). Compared with the control, the coated trace element groups significantly increased the laying rate, eggshell strength and yolk color, and decreased the cracked egg rate and feed-to-egg ratio (*p* < 0.05). Group B had a lower cracked egg rate and a higher egg shape index than group C. Regarding eggshell ultrastructure, group B had a greater effective layer thickness and proportion and a lower mammillary layer thickness and proportion (*p* < 0.05), while group C presented increased mammillary width. The coated trace elements increased the deposition of Mn, Zn, and Se in eggs and increased serum GSH-Px and Cu-Zn SOD activities and the T3 concentration. No significant differences in hatching performance were observed among the groups. For progeny, groups B and C had higher birth weight, pectoralis muscle weight, and liver weight, as well as improved serum antioxidant indices and intestinal morphology (*p* < 0.05). The mRNA expression of intestinal tight junction genes was upregulated in group C (*p* < 0.05) and showed a modest increase in group B. In conclusion, the coated inorganic compound trace elements, when used at reduced inclusion rates, replaced the conventional uncoated inorganic premix without compromising production performance and improved several performance outcomes. The 500 mg/kg inclusion level provided the most balanced improvements in breeder performance and progeny quality while reducing feed costs. These findings suggest that coated mineral technology may sustain productivity at a substantially lower mineral input and may benefit progeny by increasing egg mineral deposition and supporting intestinal development, thereby providing a more sustainable mineral-feeding strategy for breeder hens.

## Simple Summary

Traditional inorganic trace mineral additives widely used in the feed industry have low bioavailability and pose a high risk of environmental contamination. Therefore, there is an urgent need to develop novel additives that are inexpensive, efficient, and less polluting. Coated inorganic compound trace elements can provide controlled intestinal release and protect labile nutrients such as vitamins, indicating promising potential for practical application. This study evaluated graded levels of a coated inorganic compound trace elements as lower-inclusion replacements for an uncoated inorganic trace mineral premix and assessed their effects on breeder hen performance and progeny development. A total of 360 healthy, 40-week-old Hy-Line Brown layer breeder hens were randomly assigned into three groups: the control group (2000 mg/kg inorganic trace elements), the low-dose coated group (500 mg/kg) and the high-dose coated group (1000 mg/kg). The formal feeding trial lasted 10 weeks. In conclusion, the coated inorganic compound trace elements, when included at reduced levels, replaced the conventional uncoated inorganic premix without impairing laying performance, eggshell quality, or progeny development. Overall, the 500 mg/kg inclusion level showed greater economic potential and may represent an effective mineral-feeding strategy for breeder hens.

## 1. Introduction

Modern livestock farming is rapidly advancing toward intensification and standardization. Simultaneously improving production efficiency, product quality, and economic returns while maintaining stable output has become a major goal of modern animal production. Rational dietary trace element supplementation is critical for optimizing the growth performance and product quality of livestock and poultry. Trace elements participate in a wide range of physiological processes in livestock and poultry, including growth and development, nutrient metabolism, neural regulation, immune responses, and embryonic development. Maternal trace mineral nutrition can influence progeny physiology through egg-mediated nutrient transfer [1]. As specialized breeding birds, breeder hens must both maintain their own metabolic homeostasis and provide sufficient nutrients for embryonic development and chick growth. These unique physiological traits enable breeder hens to have higher dietary trace element requirements than commercial laying hens. Trace elements are indispensable for hematopoiesis, skeletogenesis and antioxidant defense [2,3,4,5]. The demand for these physiological processes increases substantially during reproduction. Accordingly, adequate dietary provision of trace elements is required, because animals lack the capacity for endogenous synthesis of these minerals. Complex antagonistic and synergistic interactions occur among trace elements in vivo. An insufficient dietary supply or an imbalance among trace elements can induce mineral deficiencies in livestock and poultry. These deficiencies may cause anemia, growth retardation, and metabolic dysfunction and, in severe cases, death [6].

Current commercial trace element additives have distinct strengths and inherent limitations. Inorganic trace element salts are prone to moisture absorption and caking during processing and storage accompanied by poor chemical stability. Their bioavailability can be compromised by fluctuations in intestinal pH, ionic antagonism, and interactions with dietary nutrients, resulting in low utilization efficiency. Inorganic trace minerals may also interfere with the absorption and utilization of dietary lipids and vitamins, thereby reducing overall feed efficiency. Excessive supplementation of inorganic trace elements additionally increases the risk of environmental heavy metal emissions and potential food safety hazards [7]. Organic trace mineral salts have moderately greater bioavailability than their inorganic counterparts but do not completely eliminate ionic antagonism or stability limitations; therefore, improvements in nutrient absorption may remain limited [8]. Trace mineral amino acid chelates, small-peptide chelates, and nanoscale trace minerals may have greater bioavailability than conventional mineral additives. However, complex production procedures, inconsistent product quality, and high manufacturing costs restrict their large-scale use in livestock production systems. However, their complex production procedures, unstable product quality and high manufacturing costs severely restrict their large-scale application in livestock production systems [9].

Coated trace element technology has become a reliable approach to address the defects of traditional trace element additives. This technology has been widely applied in the veterinary industry with mature processing techniques and low production costs [10]. In the present study, the enteric coating used employs acid-resistant materials, including ethyl cellulose and polyacrylic resin, that polyacrylic resin to physically encapsulate the inorganic mineral particles, without altering their inorganic chemical form. This coating prevents trace elements from gastric degradation, reduces ionic antagonism in the digestive tract, improves product stability, and enables targeted delivery to the small intestine for sustained absorption, thereby substantially enhancing the bioavailability and utilization efficiency of dietary trace elements [11]. Furthermore, coating may improve the palatability of trace mineral additives, mask unpleasant tastes and odors, and reduce gastrointestinal irritation caused by the minerals. Previous studies have shown that coated trace mineral supplementation improves nutrient digestibility, product stability, and metabolic utilization and may enhance growth performance, antioxidant capacity, and meat quality in broilers [12,13]. The incorporation of coated trace elements in feed provides a feasible solution to resolve the common drawbacks of traditional trace element additives, including low bioavailability, poor stability, ionic toxicity, and poor economic performance. Moreover, by improving absorption efficiency, coated trace elements reduce mineral excretion, thereby alleviating heavy metal accumulation in soil and water bodies.

Despite these promising findings in broilers, limited information is available regarding the efficacy of coated compound trace elements in laying breeder hens, particularly with respect to their impact on egg quality, eggshell ultrastructure, and maternal programming of offspring development. It also remains unclear whether coated compound trace elements can replace an uncoated inorganic premix at a lower inclusion level without compromising performance. Therefore, we hypothesized that dietary supplementation with coated inorganic compound trace elements, by virtue of their enteric protection and targeted intestinal release, would improve breeder performance and offspring development while reducing mineral supplementation without compromising productivity, thereby achieving economic and environmental benefits.

The present study used graded levels of a coated compound of inorganic trace elements to replace a high-inclusion inorganic premix in breeder hen diets. This substitution strategy was evaluated using laying performance, egg quality, eggshell ultrastructure, trace mineral deposition, serum antioxidant status, and metabolic indices. The study also examined the effects of maternal supplementation on progeny growth and intestinal development and evaluated the more effective inclusion level of the coated compound trace elements. This study aimed to develop a mineral-feeding strategy that improves breeder hen performance, reduces feed costs, and potentially limits trace mineral pollution, thereby promoting the sustainable development of green and healthy livestock production.

## 2. Materials and Methods

### 2.1. Ethics Statement

The management and care procedures for the animals used in this study were approved by the Institutional Animal Care and Use Committee of the Northeast Agricultural University (Approval No.: NEAU-20251018-2, 18 October 2025).

### 2.2. Animals, Diets, and Experimental Design

This study used a single-factor experimental design. A total of 360 healthy, 40-week-old Hy-Line Brown breeder hens, with similar baseline body weights and laying rates, were randomly assigned to three treatments. Each treatment comprised six replicate pens of 20 hens. Birds in the control group received a basal diet supplemented with 2000 mg/kg of an uncoated inorganic trace element premix. The two experimental groups received the same basal diet supplemented with 500 or 1000 mg/kg of the coated inorganic trace element premix, respectively, as lower-inclusion alternatives to the uncoated premix used in the control group. The trace minerals in all three treatments were inorganic salts; the treatments differed only in coating status and total premix inclusion level. The inorganic coated compound trace element products were provided by Woori Biotechnology Shanghai Co., Ltd., Shanghai China. All experimental diets were formulated to be isonitrogenous and isoenergetic, with differences limited only to the supplemental forms and inclusion levels of trace elements. The trial lasted 11 weeks and comprised a 1-week adaptation period followed by a 10-week formal feeding period. All hens were offered the basal diet during the early adaptive phase. The experimental diets were introduced gradually from day 5 of the adaptation period by replacing 25% of the basal diet each day. Full dietary transition was completed on the eighth day, when the formal trial commenced. Artificial insemination was conducted routinely at 13:00 h throughout the formal trial period. Semen was collected from healthy Hy-Line Brown male breeders of the same age using the dorsoventral massage method. The collected semen was pooled immediately and maintained at 37 °C in a water bath. Insemination was completed within 30 min of collection, and each hen received 0.05 mL of semen deposited at a depth of approximately 2–3 cm. Hatching eggs were collected by treatment when the hens reached 51 weeks of age. Twenty qualified hatching eggs were obtained from each replicate, yielding 120 incubation eggs per treatment.

The coated inorganic compound trace elements utilized in this trial were produced through a multilayer coating technique. Briefly, the raw trace element powders were homogenously mixed with starch and dextrin binders before granulation. The formed granules were coated with ethyl cellulose to achieve controlled trace element release in the intestinal tract. A fluidized bed coating system was further applied to wrap the outer layer with polyacrylic resin, which endowed the product with excellent acid resistance and enteric solubility. The final product had a particle size of 0.55–1.70 mm, and the coating accounted for approximately 5–13% of the total product weight. The contents of specific trace elements in the coated product are presented in Table 1. The release profile of this coated product is shown in Table A1 in Appendix A.

The basal diet was formulated in accordance with practical commercial breeding production criteria. The detailed ingredient composition and nutrient levels of the basal diet are presented in Table 2, and the specific trace element profiles of compound additives in different treatments are listed in Table 3. All experimental diets maintained consistent nutritional parameters, and variations among groups were solely derived from different trace element supplementation regimens. The daily trace element intake per chicken in each group is provided in Appendix A Table A1. Compared with the control group (12 RMB/tonne), the trace element premix costs for groups B and C were reduced to 4.75 and 9.5 RMB/tonne of feed, respectively (estimated based on market prices as of July 2026).

### 2.3. Laying Performance

Laying performance was routinely monitored starting at 41 weeks of hen age. Daily egg production and individual egg weight were recorded on a per-replicate basis, and all harvested hatching eggs were categorized into qualified and unqualified grades. Laying rate, qualified egg rate, and cracked egg rate were calculated weekly based on daily production data. Mean egg weight was determined biweekly throughout the trial period. Feed consumption was measured regularly to compute average daily feed intake and feed-to-egg ratio. Total feed intake and total egg weight of each replicate across the entire experiment were further summarized to obtain the overall average egg weight, average daily feed intake, and feed-to-egg ratio for the whole trial.

### 2.4. Egg Quality Parameters

Egg quality evaluation was performed at the end of the 45th and 48th weeks of age. Ten intact fresh eggs were randomly selected from each replicate for subsequent quality determination. Yolk color, albumen height, and Haugh unit were measured by a multifunctional egg quality analyzer (EA-01, Orka Food Technology, Binyamina, Israel). Egg shape index was determined with a professional egg shape index tester (NFN385, Fujihira Industry Co., Ltd., Tokyo, Japan). Eggshell breaking strength was detected via an Egg Force Reader (EFR, Orka Food Technology, Binyamina, Israel). For eggshell thickness assessment, three representative positions including the egg blunt end, sharp end, and equator were measured using a vernier caliper, and the average value of the three measurements was defined as the final eggshell thickness of each sample.

### 2.5. Eggshell Ultrastructure

Eggshell ultrastructural analysis was conducted at the end of the 48th week of age. Ten intact eggshell specimens were randomly collected per replicate. The ultrastructural morphology of eggshells was observed using a scanning electron microscope FEI Quanta 600 (FEI Company, Hillsboro, OR, USA) at 200× magnification. Key ultrastructural indices including effective layer thickness, mammillary layer thickness, and mammillary width were quantified. The average values of ten eggshell samples from each replicate served as the replicate statistical data. The relative proportions of the effective layer and mammillary layer were further calculated according to the corresponding thickness parameters.

### 2.6. Trace Element, Calcium, and Phosphorus Contents in Egg White, Yolk, and Eggshell

At 45 and 48 weeks of age, three eggs were randomly selected from each replicate for nutrient analysis; these time points represented the transition from the middle to the late laying period. Egg yolk and albumen were precisely separated, and eggshell samples from the same replicate were fully mixed to prepare composite samples. All samples were used for the determination of iron, copper, manganese, zinc, selenium, calcium, and phosphorus contents in egg yolk, albumen, and eggshell. Fresh yolk and albumen samples were freeze-dried to a constant weight using a benchtop Quorum K750X freeze-dryer (Quorum Technologies, Lewes, UK) and then ground into a uniform powder. Trace element concentrations were determined by Agilent 7800 inductively coupled plasma mass spectrometer (Agilent Technologies, Santa Clara, CA, USA). Calcium content was measured via potassium permanganate titration, while phosphorus content was quantified using the ammonium vanadate–molybdate colorimetric method.

### 2.7. Hatching Performance

Qualified hatching eggs were collected when breeder hens reached 50 weeks of age. Twenty eligible eggs were selected per replicate, yielding 120 incubation eggs per treatment group. All eggs were stored in a dark constant-temperature environment at 10 °C. After the completion of sample collection, all eggs were incubated synchronously in an FT-ZF10 automatic incubator (Fangtong Electronics, Nanchang, China). The incubation temperature was 37.5 °C, and relative humidity was maintained at 55–60%. The eggs were turned automatically every 2 h until day 18, when turning was stopped 3 days before hatching. The eggs were candled on day 15 of incubation to count fertile eggs and calculate the fertility rate. Upon the completion of hatching, the total number of hatched chicks and healthy chicks were recorded for each replicate to determine hatchability and healthy chick rate.

### 2.8. Parental and Progeny Sample Collection and Processing

At 49 weeks of age, one healthy breeder hen with a body condition representative of its replicate was selected from each replicate, and blood was collected from the brachial vein. For progeny sample collection, three one-day-old chicks were randomly euthanized per replicate to obtain blood and tissue samples. All blood samples were centrifuged at 3000 rpm for 20 min at 4 °C. The isolated serum was transferred into 1.5 mL cryotubes and stored at −20 °C for subsequent biochemical detection. Intestinal segments of 2 to 3 cm in length were harvested from the duodenum, jejunum, and ileum of euthanized chicks. Intestinal luminal contents were gently rinsed with sterile normal saline. Partial intestinal tissues were fixed in 4 percent paraformaldehyde solution for subsequent histomorphological analysis. Fresh jejunal tissue samples were placed in sterile cryogenic tubes, immediately snap-frozen in liquid nitrogen, and stored at −80 °C for long-term preservation and downstream molecular assays.

### 2.9. Offspring Growth Performance Parameters

Hatching eggs were collected at the early stage of the 50th week of age. Twenty qualified eggs per replicate were individually weighed to calculate the average incubation egg weight. The egg incubation procedure was performed in accordance with the aforementioned methods. Newly hatched chicks were weighed on a per-replicate basis to record initial birth weight. Three one-day-old chicks with body weight close to the replicate average were selected and euthanized. Breast muscle, leg muscle, and liver tissues were dissected and weighed to evaluate the growth and development status of progeny muscles and visceral organs.

### 2.10. Serum Antioxidant and Metabolic Parameters

Serum concentrations and activities of triiodothyronine (T3), tetraiodothyronine (T4), glutathione peroxidase (GSH-Px), and copper–zinc superoxide dismutase (Cu-Zn SOD) were determined using commercial assay kits purchased from Nanjing Jiancheng Bioengineering Institute, Nanjing, China. All experimental operations were strictly implemented in compliance with manufacturer standard protocols. The absorbance of each sample was measured using a multifunctional microplate reader (GENios, TECAN, Männedorf, Switzerland) to calculate corresponding serum metabolic and antioxidant parameters.

### 2.11. Intestinal Histomorphology

Intestinal tissues fixed in 4 percent paraformaldehyde solution for 24 h were subjected to gradient ethanol dehydration and paraffin embedding. Continuous tissue sections were prepared and stained with hematoxylin and eosin for histological observation. Representative microscopic fields were selected and photographed under an optical microscope. Villus height and crypt depth were measured using ImageJ 2.7.0 software, and the villus height to crypt depth ratio was calculated accordingly. Five parallel measurements were performed for each tissue section, and the average value was adopted as the final morphological data of the sample.

### 2.12. Offspring Jejunal Mucosal Barrier-Related Gene Expression

Frozen jejunal tissue samples were fully ground under liquid nitrogen conditions. Total RNA was extracted from the tissue samples using TRIzol reagent, and reverse transcription was performed using the PrimeScript RT Reagent Kit (TaKaRa, Kusatsu, Shiga, Japan). The target genes included tight junction-related genes *Occludin* and *ZO-1*, as well as the intestinal mucosal barrier functional gene *MUC2*. Chicken GAPDH was used as the internal reference gene. Quantitative real-time polymerase chain reaction was carried out using SYBR Premix Ex Taq kit (TaKaRa, Kusatsu, Shiga, Japan). The relative expression levels of target genes were calculated via the 2^−ΔΔCt^ method based on real-time fluorescence amplification curves. All primer sequences used in this experiment are listed in Table 4.

### 2.13. Statistical Analysis

The replicate of 20 breeder hens was considered the experimental unit. Multiple eggs or birds sampled within each replicate were treated as subsamples and averaged to obtain one replicate value (n = 6 per treatment). Treatment was specified as a categorical fixed factor rather than a continuous dose variable. Residual normality and homogeneity of variance were assessed using Q–Q plots, the Shapiro–Wilk test, and Levene’s test, respectively. Overall continuous outcomes measured once were analyzed by one-way ANOVA, followed by Tukey-adjusted pairwise comparisons. Welch’s ANOVA or the Kruskal–Wallis test was used when the relevant assumptions remained unmet after transformation. Repeated continuous outcomes, including weekly egg weight, feed intake, feed-to-egg ratio, and variables measured at both 45 and 48 weeks, were analyzed using linear mixed-effect models with treatment, time, and their interaction as fixed effects and replicate as a random effect. Repeated proportional outcomes, including laying rate, qualified egg rate, and cracked egg rate, were analyzed from their numerators and denominators using binomial generalized linear mixed models with treatment, time, and their interaction as fixed effects and replicate as a random effect. Fertility, hatchability, and healthy chick rate were analyzed similarly using their corresponding event counts and denominators. Two pre-specified contrasts were evaluated: the inorganic control versus the mean of the two coated mineral treatments, and 500 versus 1000 mg/kg inorganic coated compound trace minerals. No linear or quadratic dose–response contrast across the three treatments was performed because the control differed from the coated treatments in both mineral source and premix inclusion level. Results are presented as estimated marginal means and SEM. Statistical significance was set at *p* < 0.05. Analyses were performed using IBM SPSS Statistics 29.0 (IBM Corp., Armonk, NY, USA).

## 3. Results

### 3.1. Laying Performance

Table 5 illustrates laying rate, qualified egg rate and cracked egg rate in 40–50-week-old layer breeders fed diets supplemented with coated inorganic compound trace elements. The detailed weekly data are shown in Table A2 of the Appendix A. Over the 10-week formal experimental period, groups B and C had higher laying rates and lower cracked egg rates than group A (*p* < 0.05). No significant difference in laying rate was found between group B and group C. Group B had a lower cracked egg rate than group C (*p* > 0.05).

Table 6 presents average egg weight, average daily feed intake and feed-to-egg ratio. The detailed weekly data are shown in Table A3 of the Appendix A. Groups B and C had lower feed-to-egg ratios during the 1–10-week formal experimental period (*p* < 0.05). Average egg weight and average daily feed intake showed no significant differences among all treatments (*p* > 0.05). No significant differences were detected between group B and group C (*p* > 0.05).

### 3.2. Egg Quality

Table 7 shows egg quality indices of layer breeders at 45 and 48 weeks of age. At 45 weeks of age, eggshell strength and yolk color in groups B and C were superior to those in group A (*p* < 0.05). Group C had a better egg shape index and group B had a deeper yolk color. No significant differences were observed among groups for most indices at 48 weeks of age (*p* > 0.05).

### 3.3. Eggshell Ultrastructure

**Figure 1:**
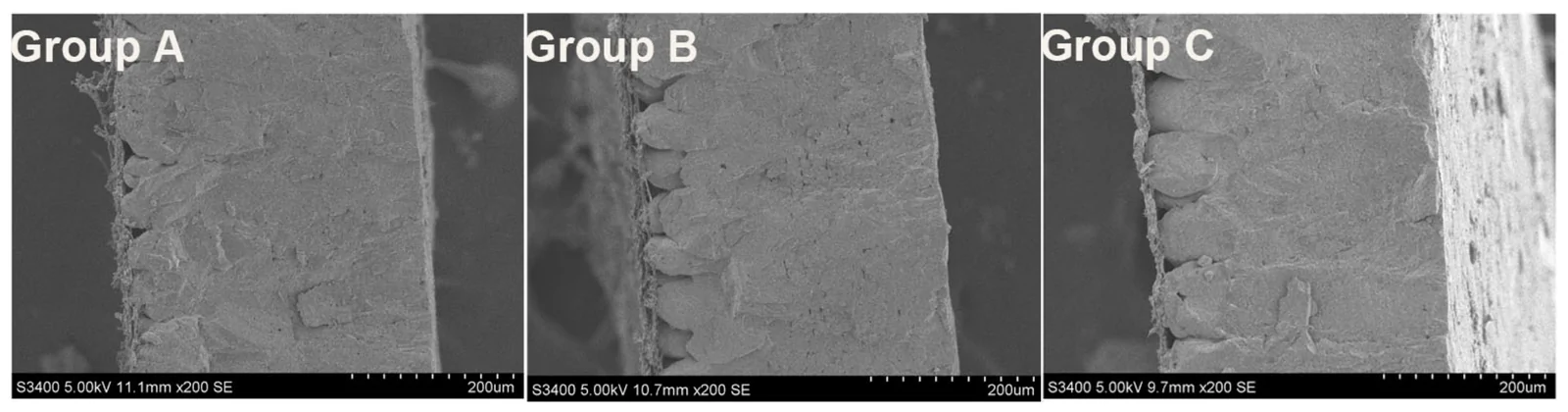
Scanning electron microscope photos of eggshell of 48-week-old eggs.

Figure 1 displays scanning electron micrographs of eggshell ultrastructure from 48-week-old layer breeders. The mammillae in groups B and C were more uniform in size and more closely arranged than those in group A. Group C had a wider mammillary diameter.

Table 8 presents eggshell ultrastructural characteristics of 48-week-old layer breeders. Group B had greater thickness and proportion of the effective layer as well as lower thickness and proportion of the mammillary layer compared with group A (*p* < 0.05). Group C showed increased mammillary width.

### 3.4. Trace Element, Calcium, and Phosphorus Contents in Eggs at 45 Weeks of Age

Table 9 shows concentrations of trace elements, calcium and phosphorus in eggs from 45-week-old layer breeders. Eggshell zinc and manganese contents were higher in group B than in group A (*p* < 0.05). Yolk selenium content was higher in groups B and C (*p* < 0.05). Albumen selenium content was higher in groups B and C and albumen zinc content was lower in group C (*p* < 0.05).

### 3.5. Trace Element, Calcium, and Phosphorus Contents in Eggs at 48 Weeks of Age

Table 10 lists trace element, calcium and phosphorus levels in eggs from 48-week-old layer breeders. Eggshell manganese, zinc and selenium contents were higher in groups B and C than in group A (*p* < 0.05). Yolk manganese and selenium contents were higher in groups B and C (*p* < 0.05). Albumen selenium content was higher in group C than in group A (*p* < 0.05).

### 3.6. Serum Antioxidant and Metabolic Parameters of Breeder Hens

Table 11 presents serum biochemical indices of 49-week-old layer breeders. Activities of glutathione peroxidase and copper–zinc superoxide dismutase and serum triiodothyronine levels were higher in groups B and C than in group A (*p* < 0.05). Copper–zinc superoxide dismutase activity was slightly higher in group B than in group C. Other parameters showed no significant differences between group B and group C (*p* > 0.05).

### 3.7. Hatchability Performance

Table 12 shows hatching performance indices of eggs from 49-week-old layer breeders. The fertility rate, hatchability and healthy chick rate exhibited no significant differences among all groups (*p* > 0.05).

### 3.8. Offspring Growth Performance

Table 13 presents the growth performance of newly hatched progeny chicks. Birth weight, breast muscle weight and liver weight of chicks from groups B and C were higher than those of chicks from group A (*p* < 0.05).

### 3.9. Offspring Serum Antioxidant and Metabolic Parameters

Table 14 shows serum biochemical indices of one-day-old progeny chicks. Serum glutathione peroxidase activity was higher in group C than in group A (*p* < 0.05). Copper–zinc superoxide dismutase activity and triiodothyronine levels were higher in groups B and C than in group A (*p* < 0.05).

### 3.10. Intestinal Morphology of Newly Hatched Offspring

**Figure 2:**
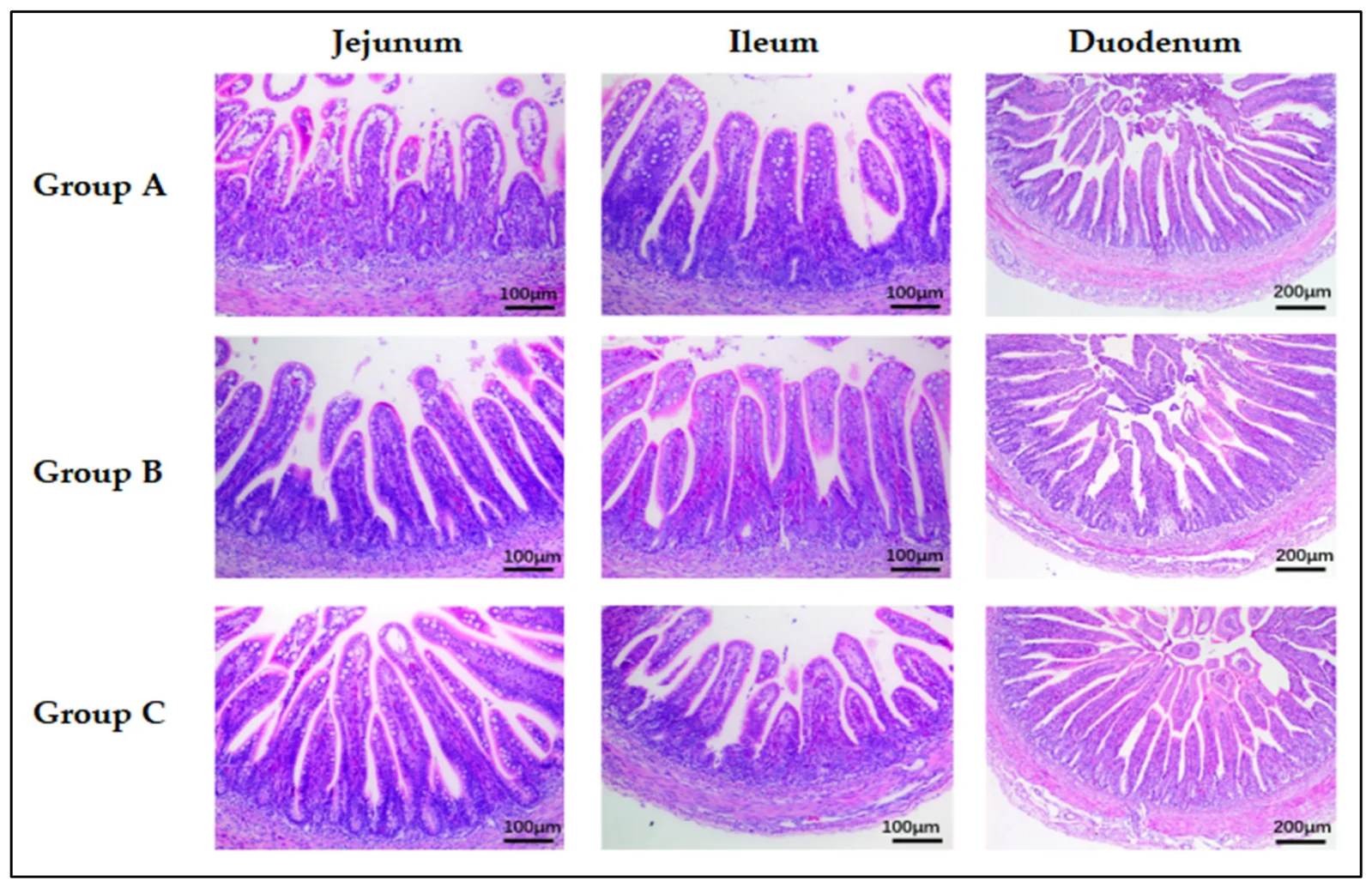
HE staining photos of jejunum, ileum and duodenum sections of newborn offspring of egg breeder hens (jejunum, ileum × 200, duodenum × 100).

Figure 2 and Table 15 show the effects of graded inclusion levels of the coated inorganic compound trace elements as partial substitutes for the uncoated inorganic trace minerals. Villus height and the ratio of villus height to crypt depth were higher and crypt depth was lower in groups B and C across the duodenum, jejunum and ileum (*p* < 0.05).

### 3.11. Relative Expression Levels of Jejunal Intestinal Barrier-Related Genes in Offspring

**Figure 3:**
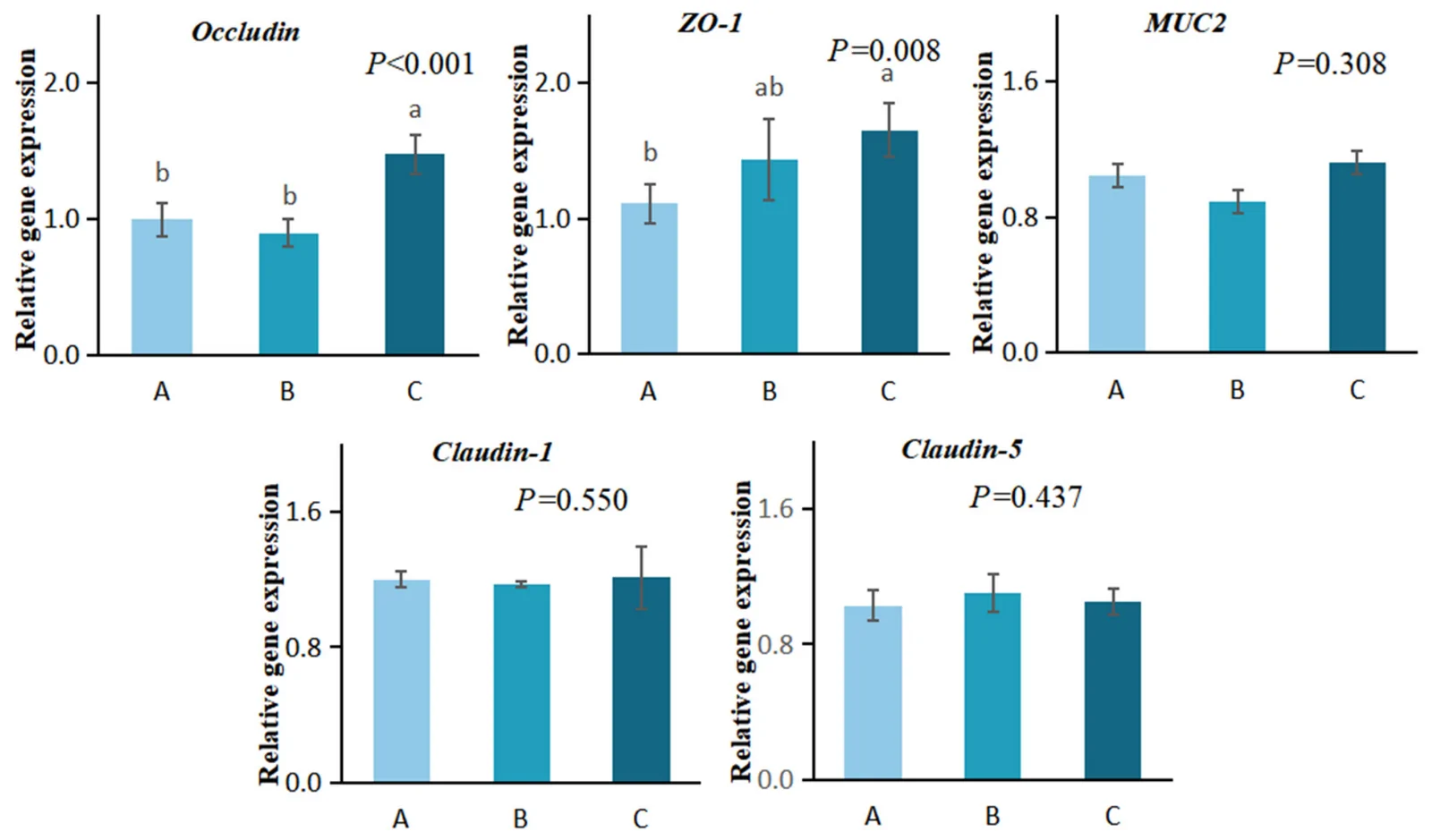
Effect of the coated inorganic compound trace elements on the relative expression of jejunal intestinal barrier-related genes in newborn offspring of egg breeder hens. ^a, b^ Means in the column without common superscripts are significantly different (*p* < 0.05). A, inorganic trace elements; B, 500 mg/kg coated inorganic compound trace elements; C, 1000 mg/kg coated inorganic compound trace elements.

Figure 3 shows the relative expression of intestinal barrier-related genes in the jejunum of newly hatched chicks. The mRNA expression of *Occludin* and *zonula occludens 1* was upregulated in group C compared with group A and group B (*p* < 0.05). The expression of these two genes slightly increased in group B relative to group A (*p* < 0.1). The mRNA levels of *mucin 2*, *Claudin-1* and *Claudin-5* showed no significant differences among the three groups (*p* > 0.05).

## 4. Discussion

Laying performance is a core indicator for evaluating production efficiency during layer breeder rearing. Dietary nutrient supply represents one critical factor regulating the laying performance of breeder hens. In our experiment, the laying performance of the experimental groups was superior to that of the inorganic control group, mainly in terms of laying rate, feed conversion ratio, and egg quality. These findings are consistent with previous reports that amino acid-complexed minerals improve laying performance in poultry and suggest that the coated inorganic trace mineral premix may have similar effects [14,15].

Egg quality is a key trait for assessing the performance of layer breeders. Common evaluation indices include egg shape index, eggshell thickness, eggshell strength, albumen height, yolk color, and Haugh unit. These parameters comprehensively reflect shell firmness, egg freshness and the quality of albumen and yolk in hatching eggs [16,17,18]. The improvement in eggshell quality, particularly the increased eggshell strength and altered ultrastructure in the low-dosage group, reflects the critical roles of zinc and manganese in shell formation. Zinc participates in carbonic anhydrase activity, which influences carbonate ion availability for calcium carbonate crystallization, while manganese regulates glycosaminoglycan synthesis in the shell gland, affecting mammillary layer organization [19,20,21]. The eggshell consists of cuticle, vertical crystal layer, palisade layer, mammillary layer and inner shell membrane from the outer surface to the inner side. The first three layers are collectively defined as the effective layer of eggshell [22]. The observed reduction in mammillary layer thickness with increased effective layer proportion in the low-dosage group is mechanistically consistent with improved crystal nucleation and growth patterns, as a thinner mammillary layer with more uniform mammillae is typically associated with enhanced shell strength [23]. Notably, the high-dosage group showed increased mammillary width but no comparable ultrastructural improvement, suggesting an optimal manganese level for mammillary organization beyond which additional supplementation provides no further benefit, possibly due to saturation of transport mechanisms or altered glycosaminoglycan composition. Earlier studies have reported that partial replacement of inorganic trace elements with organic compound trace elements at reduced dosages can optimize eggshell ultrastructure in laying hens [24,25]. The present results are generally consistent with these conclusions, as supplementation with different forms of trace elements in layer diets significantly increased eggshell thickness and strength compared with the control group. In addition, trace elements can influence antioxidant enzyme activity, which is a key factor in maintaining hatching egg freshness and is associated with Haugh unit and albumen quality, while also regulating uterine status and thereby affecting egg quality [26,27]. In the present study, egg shape index and albumen height in the experimental groups showed non-significant increasing trends compared with the control group, which is consistent with previous reports [28,29]. The low-dosage coated group had higher eggshell strength and superior yolk color, whereas the high-dosage group showed significant reductions in egg shape index and yolk color, with other indices showing similar trends to the low-dosage group. The lower yolk color score in the high-dose group may have resulted from antagonistic interactions between trace minerals and lipids after intestinal release, which could have interfered with lutein deposition [30]. These results suggest that the coated compound inorganic trace elements may enhance the utilization efficiency of trace elements in layer breeder hens, likely attributable to their slow-release properties, and the effects appear to be dose-dependent.

Differences in trace element contents across various egg fractions at different sampling time points further support the above conclusion. The elevated selenium content in yolk and albumen is of particular significance, as selenium plays a crucial role in supporting embryonic antioxidant defense and development [27]. The observation that egg mineral concentrations were generally higher at 48 weeks than at 45 weeks suggests a cumulative deposition effect over time, which may reflect a gradual saturation of egg mineral content as supplementation continues. In addition, we observed that, although trace element levels in eggs from both treatment groups were generally higher than those in the control group, the high-dosage group exhibited lower levels than the low-dosage group, which may be attributable to antagonistic interactions among trace elements. Min et al. [31] and Domel et al. [32] found that organic or chelated trace elements could increase trace element deposition in eggshells. Dong et al. [33] also reported that organic trace element chelates promoted trace element accumulation in egg yolk. Collectively, coated compound trace elements may have greater bioavailability than traditional inorganic sources and may improve laying performance, eggshell ultrastructure, and overall egg quality. Ruangpanit et al. [34] demonstrated that reducing the supplemental dose of coated trace minerals can elevate mineral utilization efficiency, which is broadly consistent with our current results. Overall, the low-dose treatment produced a more balanced response than the high-dose treatment.

The reproductive performance of breeder hens determines the quantity and quality of commercial progeny and serves as an important criterion for evaluating production performance and economic benefits. Maternal nutritional status affects nutrient deposition in oocytes and further influences early embryonic development and subsequent growth of offspring. Previous studies have demonstrated that trace elements regulate reproductive performance and early embryonic neural development in chickens [35,36]. Despite the increased mineral content in eggs, hatchability did not differ significantly among groups, suggesting that the basal inorganic supplementation already met the minimum requirements for embryonic survival, and additional mineral enrichment above this threshold did not further enhance hatchability. However, the greater hatch weight, breast muscle weight, and liver weight in progeny from the treatment groups indicate that the excess minerals deposited in eggs supported embryonic or early post-hatch development, rather than merely affecting hatchability itself. This maternal programming effect may involve selenium-dependent antioxidant protection during late embryogenesis and zinc-mediated regulation of protein synthesis and degradation pathways in muscle, as both elements are critical for muscle development and metabolic programming [27,37,38,39]. In the present study, improvements in progeny traits were more pronounced in the high-dose group, whereas breeder performance was similar between the two coated-premix groups. This pattern suggests that the maternal intake required to alter egg mineral deposition and progeny traits may differ from that required to affect breeder performance. Morris et al. [40] observed numerical increases in fertilization rate, hatchability and healthy chick rate in hens fed diets supplemented with amino acid chelated compound trace elements without significant statistical differences. Their observations match the results obtained in the present study. Wang et al. [41] demonstrated that chicks hatched from breeder hens receiving collagen peptide chelated trace elements had higher hatching body weight and breast muscle percentage at 14 days of age. Similar results have also been reported by Urso et al. [42]. In conclusion, coated inorganic compound trace elements may improve the comprehensive reproductive performance of layer breeders, likely by promoting the deposition of zinc, selenium, and other trace elements in eggs.

Serum antioxidant capacity and metabolic indices reflect redox and metabolic status in animals. These physiological traits are regulated by trace elements and are closely associated with production performance. Glutathione peroxidase (GSH-Px) and copper–zinc superoxide dismutase (Cu-Zn SOD) are major antioxidant enzymes that eliminate hydrogen peroxide and lipid peroxides, protecting cells and tissues from oxidative damage [12]. The elevated serum activities of GSH-Px and Cu-Zn SOD in both treatment groups directly reflect the roles of selenium as a structural component of GSH-Px and copper/zinc as cofactors for SOD [43,44]. The concurrent increase in T3 levels in the treatment groups can be mechanistically attributed to selenium-dependent deiodinase activity, as type I and type II iodothyronine deiodinases are selenoproteins that catalyze the conversion of T4 to active T3 [45]. This selenium-mediated enhancement of thyroid hormone metabolism may contribute to improved metabolic efficiency and growth performance, as T3 is a key regulator of basal metabolic rate and protein synthesis. Nie et al. [46] reported that dietary supplementation with coated inorganic compound trace elements increased serum total antioxidant capacity, GSH-Px, and SOD activities in broilers. Mohamed et al. [47] found that compound trace element additives induced an increasing trend in serum T3 levels, with no significant effect on T4 levels in broilers. Wang et al. [48] noted that maternal supplementation with organic compound trace elements improved plasma T-AOC, SOD, and Cu-Zn SOD activities in broiler offspring. The results of the present study are generally consistent with these findings. However, we observed that the high-dosage group showed greater effects in progeny, and the optimal dosage of coated inorganic compound trace elements differed between breeder hens and their offspring. This pattern may reflect maternal transfer of dietary minerals. The low dose may have met the metabolic demands of the breeder hens but produced less mineral enrichment of the eggs, whereas the high dose may have increased mineral deposition in hatching eggs and thereby influenced embryonic development and the antioxidant and metabolic status of the progeny. In conclusion, maternal dietary supplementation with coated inorganic compound trace elements can simultaneously improve the antioxidant capacity and metabolic status of both breeder hens and their progeny.

The intestine is the primary organ responsible for nutrient absorption in poultry. Intestinal morphology is a critical index for evaluating animal growth performance. Villus height, crypt depth and the ratio between these two parameters are classic morphological indicators. They can reflect the digestive and absorptive capacity as well as developmental status of intestinal tissues [49]. The improved intestinal morphology in progeny, characterized by increased villus height and enhanced villus-to-crypt ratio, reflects enhanced absorptive surface area and epithelial turnover. Since villus height correlates positively with nutrient transport capacity, these morphological changes likely contributed to improved growth performance in treatment groups [50]. The jejunum presented the most remarkable response to trace element supplementation. Variations in treatment effects among different intestinal segments may be linked to the sequential developmental characteristics of intestinal tissues [51]. Gopi et al. [52] found that supplemental organic zinc improved villus height and the ratio of villus height to crypt depth in different intestinal segments of breeder hens but did not alter the intestinal morphology of their offspring. Chen et al. [53] evaluated the effects of organic compound trace elements on late laying hens and reported that 450 mg/kg organic compound trace elements increased duodenal villus height and the ratio of villus height to crypt depth. Malheiros et al. [54] observed superior performance from coated trace minerals versus uncoated ones at the same inclusion level, with low doses showing advantages over high doses. The results of the present study are in general agreement with their reports.

The intestinal mucosal barrier can be functionally classified into mechanical, chemical, immune and biological barriers [55]. Occludin, zonula occludens-1 (ZO-1) and claudins are major tight junction proteins that participate in the formation of intestinal tight junctions, enhance intercellular adhesion and maintain the stability of intestinal mechanical barrier [56,57]. The significant upregulation of occludin and ZO-1 expression in the high-dose group, together with the modest increase in ZO-1 expression in the low-dose group, suggests that maternal mineral status may influence genes related to intestinal barrier integrity, potentially through epigenetic or endocrine-mediated programming during embryonic development. Mucin 2 (MUC2) is a type of mucin secreted by intestinal goblet cells and is involved in intestinal immunity and digestion; its expression level reflects the functional status of the intestinal chemical barrier [58]. The absence of a significant change in MUC2 expression suggests that the chemical barrier may be less responsive to maternal mineral status than the mechanical barrier. Chen et al. [59] demonstrated that graded levels of coated inorganic compound trace elements upregulated the mRNA expression of *Occludin*, *ZO-1* and *Claudin-1* in broiler jejunum, and the dosage of 600 mg/kg produced the optimal effect. Shao et al. [60] and Nie et al. [61] also confirmed that compound trace elements enhanced the expression of intestinal tight junction proteins in broilers. Similar results have been observed in pigs fed coated inorganic compound trace elements [62]. Roque et al. [63] found that broiler progeny from breeders fed methionine chelated trace elements had higher jejunal *MUC2* expression compared with the group receiving inorganic trace elements. No significant difference in *MUC2* mRNA expression was detected among groups in the present study. This discrepancy may be related to the properties of coating materials used in the experiment. In conclusion, coated inorganic compound trace elements exert positive effects on promoting intestinal development and upregulating intestinal tight junction protein expression in progeny of layer breeders.

There are several caveats associated with the present work. Evaluations were limited to two inclusion rates of coated inorganic compound trace elements, which prevents us from characterizing the complete dose–response profile for this feed additive. All observations were obtained from Hy-Line Brown hens within a single experimental environment, and caution should therefore be exercised when extending these findings to alternative genetic stocks or different management practices. While improvements in production performance and intestinal phenotypes were recorded, we did not probe key gene profiles linked to intestinal mineral uptake and transport, leaving the underlying molecular regulatory mechanisms unelucidated. Potential environmental gains presumed from lowering inorganic trace mineral inputs could not be firmly verified, given the absence of direct measurements on mineral outputs in excreta. Additionally, the economic value under commercial conditions remains to be established because a systematic cost–benefit analysis was not performed in the present trial.

Future studies with a wider range of dosages, multiple breeds, long-term production cycles, and direct measurement of fecal mineral output would help to address these limitations and further validate the application of coated inorganic compound trace elements in layer breeder production.

## 5. Conclusions

Traditional uncoated inorganic trace mineral additives widely used in the feed industry have low bioavailability and pose a risk of environmental contamination. Therefore, there is an urgent need to develop low-cost, high-efficiency and low-pollution novel additives. Coated inorganic compound trace elements can reduce ionic antagonism in the digestive tract, provide controlled intestinal release, and protect active substances such as vitamins, presenting promising application prospects. This study evaluated the effects of graded levels of coated inorganic compound trace elements as a reduced substitution for traditional uncoated inorganic trace elements and assessed their effects on breeder hen performance and progeny development. A total of 360 healthy 40-week-old Hy-Line Brown layer breeders were randomly assigned into three groups: the control group (2000 mg/kg inorganic trace elements), the low-dose coated group (500 mg/kg) and the high-dose coated group (1000 mg/kg), with a 10-week trial period. Low-dose coated inorganic compound trace minerals can act as an effective low-dosage substitute for high-dose uncoated inorganic trace mineral premix, and significantly improve laying performance, eggshell ultrastructure, antioxidant status, and intestinal development of breeder hens and their progeny. The 500 mg/kg inclusion level provided the most balanced overall response and reduced premix costs, supporting its use as a lower-input mineral-feeding strategy for breeder hen production.

## Figures and Tables

**Table 1:** Content of coated inorganic compound trace elements (g/kg).

Element ^1^	Concentration
Cu	11
Fe	21
Zn	98
Mn	122
Se	0.4
I	0.8

^1^ The concentrations of the individual elements in the compound trace elements were measured values. The raw material consisted of basic copper chloride (Cu_2_(OH)_3_Cl), ferrous sulfate (FeSO_4_·H_2_O), zinc sulfate (ZnSO_4_·H_2_O), manganese sulfate (MnSO_4_·H_2_O), sodium selenite (Na_2_SeO_3_), and calcium iodate (Ca(IO_3_)_2_).

**Table 2:** Composition and nutrient levels of the basal diet (dry-matter basis, %).

Ingredients, %	Content	Nutrient Levels ^2^	Content
Corn	67.43	ME, MJ/kg	11.52
Soybean meal	21.30	Crude protein, %	14.36
Soybean oil	0.90	Ca, %	3.52
L-Met	0.06	Available phosphorus, %	0.20
Lys	0.03	Lysine, %	0.75
Limestone	8.80	Methionine, %	0.33
CaHPO_4_	0.80		
NaCl	0.17		
Choline chloride	0.07		
Premix ^1^	0.44		
Total	100.00		

^1^ Egg breeder premix per kg of diet could provide: vitamin A, 15300 IU; vitamin D3, 5610 IU; vitamin E, 93 IU; menadione, 8.5 mg; riboflavin, 26 mg; nicotinamide, 69 mg; calcium pantothenate, 26 mg; pyridoxine•HCl 8.7 mg; biotin, 0.69 mg; folic acid 7.68 mg; vitamin B12, 0.07 mg. Compound trace elements are shown in Table 3. ^2^ Nutrient levels are calculated values.

**Table 3:** Content of specific elements in compound trace elements (mg/kg).

Element ^1^	A	B	C
Cu	17.05	5.5	11
Fe	72.28	10.5	21
Zn	122.12	49	98
Mn	121.54	61	122
Se	0.33	0.2	0.4
I	1.07	0.4	0.8

^1^ Content of specific elements in compound trace elements are measured values.

**Table 4:** Primer design.

Gene	Accession No.	Primer Sequence (5′ → 3′)	Product (bp)
*GAPDH*	NM_204305.2	F:GGGCACCCATCACTATCTCTT	187
		R:TCACAAACATGGGGGCATCA	
*Occludin*	NM_205128.1	F:AGGTCTGCAACAGCATCACA	157
		R:ATGCCTTCCCAAAAAGCCCT	
*ZO-1*	XM_040680626.2	F:CCAACAGGGGCAGTCTGATG	234
		R:GAGCCCTGTGAAGAGTCACC	
*MUC2*	XM_040673077.2	F:GTGTGCCCTGATGTCACAGA	246
		R:GGCCTGAGCCTTGGTACATT	
*Claudin-1*	NM_001013611.2	F:AGAAGATGCGGATGGCTGTC	114
		R:AAGGGTCATAGAAGGCCCGA	
*Claudin-5*	NM_204201.2	F:TTGCAGGTCGCCAGAGATAC	269
		R:AGGCAAGTGCATGTTACCGA	

**Table 5:** Effects of coated inorganic compound trace elements on laying performance of 40–50-week-old egg breeder hens.

Period	Group	Laying Rate (%)	Qualified Egg Rate (%)	Cracked Egg Rate (%)
	A	80.29 ± 1.20 ^b^	94.72 ± 1.28	0.46 ± 0.12 ^a^
1~10	B	84.48 ± 1.58 ^a^	96.02 ± 0.54	0.13 ± 0.07 ^b^
	C	84.69 ± 1.22 ^a^	96.45 ± 0.59	0.32 ± 0.25 ^ab^
	*p*-value	0.038	0.373	0.094

^a, b^ Means in the column without common superscripts are significantly different (*p* < 0.05). A, inorganic trace elements; B, 500 mg/kg coated inorganic compound trace elements; C, 1000 mg/kg inorganic coated compound trace elements.

**Table 6:** Effects of coated inorganic compound trace elements on laying performance of 40–50-week-old egg breeder hens.

Period	Group	Average Egg Weight (g)	Average Daily Feed Intake (g)	Feed-To-Egg Ratio
	A	61.05 ± 0.64	118.65 ± 1.13	2.58 ± 0.09 ^a^
1~10	B	61.81 ± 0.75	118.24 ± 1.39	2.33 ± 0.06 ^b^
	C	62.52 ± 0.24	117.85 ± 1.21	2.30 ± 0.06 ^b^
	*p*-value	0.250	0.905	0.015

^a, b^ Means in the column without common superscripts are significantly different (*p* < 0.05). A, inorganic trace elements; B, 500 mg/kg coated inorganic compound trace elements; C, 1000 mg/kg coated inorganic compound trace elements.

**Table 7:** Effects of coated inorganic compound trace elements on egg quality.

Period	Group	Egg Shape Index	Eggshell Thickness (mm)	Eggshell Strength (N)	Yolk Color	Albumen Height (mm)	Haugh Unit
5	A	1.30 ± 0.01 ^a^	0.36 ± 0.01	39.64 ± 0.61 ^b^	8.18 ± 0.20 ^b^	5.11 ± 0.24	69.31 ± 2.74
	B	1.29 ± 0.01 ^ab^	0.36 ± 0.00	42.14 ± 0.33 ^a^	9.24 ± 0.11 ^a^	5.53 ± 0.20	72.62 ± 1.52
	C	1.28 ± 0.00 ^b^	0.38 ± 0.01	42.02 ± 0.89 ^a^	7.42 ± 0.24 ^c^	5.36 ± 0.36	71.60 ± 3.13
	*p*-value	0.037	0.113	0.032	0.001	0.57	0.656
8	A	1.30 ± 0.01	0.38 ± 0.01	40.78 ± 0.64	8.42 ± 0.17	5.34 ± 0.19	71.82 ± 1.72
	B	1.30 ± 0.01	0.38 ± 0.01	42.07 ± 0.49	8.16 ± 0.13	5.62 ± 0.17	74.25 ± 1.58
	C	1.27 ± 0.00	0.38 ± 0.01	41.95 ± 0.47	8.02 ± 0.14	5.47 ± 0.12	73.90 ± 1.12
	*p*-value	0.71	0.971	0.117	0.185	0.485	0.485

^a, b, c^ Means in the column without common superscripts are significantly different (*p* < 0.05). A, inorganic trace elements; B, 500 mg/kg coated inorganic compound trace elements; C, 1000 mg/kg coated inorganic compound trace elements.

**Table 8:** Effects of coated inorganic compound trace elements on the eggshell ultrastructure.

Group	Mammillary Width (μm)	Effective Layer Thickness (μm)	Mammillary Layer Thickness (μm)	Total Thickness (μm)	Effective Layer (%)	Mammillary Layer (%)
A	62.64 ± 2.18	250.16 ± 2.19	84.24 ± 1.94 ^a^	334.40 ± 2.42	74.81 ± 0.51 ^b^	25.19 ± 0.51 ^a^
B	65.27 ± 2.56	261.60 ± 3.80	77.29 ± 1.84 ^b^	338.89 ± 4.96	77.20 ± 0.38 ^a^	22.80 ± 0.38 ^b^
C	67.03 ± 1.67	262.40 ± 5.96	84.14 ± 1.08 ^a^	346.54 ± 5.49	75.69 ± 0.57 ^ab^	24.31 ± 0.57 ^ab^
*p*-value	0.383	0.118	0.018	0.197	0.015	0.015

^a, b^ Means in the column without common superscripts are significantly different (*p* < 0.05). A, inorganic trace elements; B, 500 mg/kg coated inorganic compound trace elements; C, 1000 mg/kg coated inorganic compound trace elements.

**Table 9:** Effects of coated inorganic compound trace elements on the contents of trace elements, calcium and phosphorus in 45-week-old eggs.

Item		Fe (mg/kg)	Cu (mg/kg)	Mn (mg/kg)	Zn (mg/kg)	Se (mg/kg)	Ca (%)	P (%)
Eggshell	A	47.19 ± 0.51 ^a^	0.30 ± 0.04	0.35 ± 0.02 ^b^	1.30 ± 0.06 ^b^	0.23 ± 0.14	22.91 ± 0.70	0.15 ± 0.01
	B	35.24 ± 2.37 ^b^	0.32 ± 0.05	0.58 ± 0.05 ^a^	1.60 ± 0.05 ^a^	0.31 ± 0.21	23.45 ± 0.60	0.16 ± 0.01
	C	28.89 ± 0.82 ^c^	0.32 ± 0.02	0.49 ± 0.06 ^ab^	1.12 ± 0.10 ^b^	0.07 ± 0.01	21.94 ± 0.41	0.15 ± 0.00
*p*-value		0.001	0.063	0.033	0.009	0.516	0.253	0.240
Yolk	A	138.00 ± 6.78	3.29 ± 0.12	3.35 ± 0.52	98.07 ± 2.91	2.10 ± 0.03 ^c^	0.06 ± 0.00	1.62 ± 0.06
	B	154.64 ± 5.86	3.30 ± 0.04	3.25 ± 0.03	100.90 ± 2.49	3.32 ± 0.13 ^a^	0.06 ± 0.00	1.64 ± 0.02
	C	150.23 ± 7.24	3.38 ± 0.10	2.43 ± 0.16	99.90 ± 0.93	2.68 ± 0.19 ^b^	0.06 ± 0.00	1.65 ± 0.01
*p*-value		0.263	0.756	0.153	0.688	0.002	0.729	0.820
Albumen	A	0.99 ± 0.00 ^b^	1.25 ± 0.10	0.03 ± 0.00	0.49 ± 0.00 ^a^	0.94 ± 0.04 ^b^	0.02 ± 0.00	0.13 ± 0.01
	B	1.22 ± 0.40 ^a^	1.24 ± 0.08	0.03 ± 0.00	0.33 ± 0.09 ^ab^	2.20 ± 0.25 ^a^	0.02 ± 0.00	0.13 ± 0.01
	C	0.36 ± 0.09 ^c^	1.22 ± 0.07	0.03 ± 0.01	0.26 ± 0.02 ^b^	2.20 ± 0.24 ^a^	0.02 ± 0.00	0.13 ± 0.01
*p*-value		0.001	0.967	0.83	0.058	0.007	0.454	0.859

^a, b, c^ Means in the column without common superscripts are significantly different (*p* < 0.05). A, inorganic trace elements; B, 500 mg/kg coated inorganic compound trace elements; C, 1000 mg/kg coated inorganic compound trace elements.

**Table 10:** Effects of coated inorganic compound trace elements on the contents of trace elements, calcium and phosphorus in 48-week-old eggs.

Item		Fe (mg/kg)	Cu (mg/kg)	Mn (mg/kg)	Zn (mg/kg)	Se (mg/kg)	Ca (%)	P (%)
Eggshell	A	27.21 ± 2.92 ^a^	0.25 ± 0.02	0.29 ± 0.07 ^b^	0.35 ± 0.00 ^b^	0 ^b^	22.41 ± 0.25	0.15 ± 0.00
	B	19.66 ± 0.65 ^b^	0.27 ± 0.02	0.41 ± 0.03 ^a^	0.60 ± 0.15 ^ab^	0.30 ± 0.01 ^a^	22.42 ± 0.28	0.16 ± 0.01
	C	21.18 ± 1.87 ^ab^	0.26 ± 0.02	0.36 ± 0.03 ^ab^	0.89 ± 0.07 ^a^	0.25 ± 0.01 ^a^	21.17 ± 0.61	0.15 ± 0.02
*p*-value		0.048	0.656	0.039	0.019	0.018	0.125	0.562
Yolk	A	146.49 ± 7.05	3.29 ± 0.17	2.58 ± 0.07 ^b^	97.41 ± 4.57	2.05 ± 0.09 ^c^	0.06 ± 0.01	1.64 ± 0.07
	B	142.75 ± 7.47	3.35 ± 0.09	3.29 ± 0.26 ^a^	97.58 ± 0.92	2.58 ± 0.12 ^b^	0.06 ± 0.00	1.63 ± 0.01
	C	157.06 ± 10.24	3.50 ± 0.05	3.50 ± 0.13 ^a^	106.24 ± 4.49	2.98 ± 0.05 ^a^	0.06 ± 0.00	1.69 ± 0.00
*p*-value		0.498	0.471	0.022	0.241	0.001	0.867	0.584
Albumen	A	0.41 ± 0.11	1.65 ± 0.33	0.02 ± 0.00	0.25 ± 0.02	1.44 ± 0.18 ^b^	0.03 ± 0.00	0.13 ± 0.00
	B	0.43 ± 0.16	1.07 ± 0.06	0.03 ± 0.00	0.14 ± 0.01	1.86 ± 0.14 ^ab^	0.03 ± 0.00	0.12 ± 0.01
	C	0.72 ± 0.25	1.42 ± 0.13	0.02 ± 0.00	0.27 ± 0.09	2.33 ± 0.06 ^a^	0.02 ± 0.00	0.14 ± 0.01
*p*-value		0.428	0.218	0.796	0.271	0.010	0.713	0.074

^a, b, c^ Means in the column without common superscripts are significantly different (*p* < 0.05). A, inorganic trace elements; B, 500 mg/kg coated inorganic compound trace elements; C, 1000 mg/kg coated inorganic compound trace elements.

**Table 11:** Effects of coated inorganic compound trace elements on serum antioxidation and metabolic indexes of breeder hens.

Group	GSH-Px (U/mL)	Cu-Zn SOD (U/mL)	T3 (ng/mL)	T4 (ng/mL)
A	29.00 ± 0.43 ^b^	41.86 ± 0.88 ^b^	38.07 ± 1.53 ^b^	449.46 ± 7.41
B	31.10 ± 0.17 ^a^	46.76 ± 2.67 ^ab^	45.51 ± 1.90 ^a^	462.68 ± 8.71
C	32.04 ± 0.48 ^a^	51.55 ± 0.21 ^a^	47.93 ± 1.76 ^a^	445.40 ± 5.25
*p*-value	0.001	0.004	0.005	0.253

^a, b^ Means in the column without common superscripts are significantly different (*p* < 0.05). A, inorganic trace elements; B, 500 mg/kg coated inorganic compound trace elements; C, 1000 mg/kg coated inorganic compound trace elements.

**Table 12:** Effects of coated inorganic compound trace elements on hatching performance of 50-week-old egg breeder hens.

Group	Fertility (%)	Hatchability (%)	Healthy Chick Rate (%)
A	96.00 ± 1.87	84.63 ± 5.68	97.65 ± 2.35
B	98.00 ± 1.22	88.84 ± 9.71	98.95 ± 1.05
C	94.00 ± 2.45	84.07 ± 4.66	98.75 ± 1.25
*p*-value	0.760	0.294	0.838

**Table 13:** Effects of coated inorganic compound trace elements on growth performance of offspring.

Group	Average Weight of Incubated Eggs (g)	Chick Birth Weight (g)	Breast Muscle Weight (g)	Leg Muscle Weight (g)	Liver Weight (g)
A	61.12 ± 0.52	45.22 ± 0.33 ^b^	1.13 ± 0.04 ^c^	0.78 ± 0.09	0.86 ± 0.02 ^b^
B	62.94 ± 1.72	46.39 ± 0.31 ^a^	1.45 ± 0.08 ^b^	0.88 ± 0.06	0.95 ± 0.03 ^a^
C	63.00 ± 0.34	46.44 ± 0.19 ^a^	1.73 ± 0.04 ^a^	0.81 ± 0.04	0.98 ± 0.04 ^a^
*p*-value	0.352	0.022	0.001	0.35	0.02

^a, b, c^ Means in the column without common superscripts are significantly different (*p* < 0.05). A, inorganic trace elements; B, 500 mg/kg coated inorganic compound trace elements; C, 1000 mg/kg coated inorganic compound trace elements.

**Table 14:** Effects of coated inorganic compound trace elements on serum antioxidation and metabolic indexes of newborn offspring of egg breeder hens.

Group	GSH-Px (U/mL)	Cu-Zn SOD (U/mL)	T3 (ng/mL)	T4 (ng/mL)
A	154.94 ± 3.78 ^b^	57.65 ± 1.15 ^b^	7.14 ± 0.31 ^b^	265.59 ± 4.56
B	156.81 ± 4.38 ^b^	61.62 ± 0.94 ^a^	9.91 ± 0.44 ^a^	302.92 ± 18.43
C	180.15 ± 4.44 ^a^	62.76 ± 0.92 ^a^	9.26 ± 0.81 ^a^	296.12 ± 8.04
*p*-value	0.002	0.009	0.006	0.049

^a, b^ Means in the column without common superscripts are significantly different (*p* < 0.05). A, inorganic trace elements; B, 500 mg/kg coated inorganic compound trace elements; C, 1000 mg/kg coated inorganic compound trace elements.

**Table 15:** Effects of coated inorganic compound trace elements on intestinal morphology of newborn offspring of egg breeder hens.

Item		Villus Height (VH)	Crypt Depth (CD)	VH/CD
Jejunum	A	309.15 ± 0.60 ^c^	82.98 ± 3.99 ^a^	3.74 ± 0.17 ^c^
	B	364.68 ± 6.37 ^b^	74.44 ± 2.38 ^ab^	4.90 ± 0.07 ^b^
	C	381.91 ± 1.08 ^a^	64.26 ± 5.45 ^b^	6.03 ± 0.50 ^a^
*p*-value		0.001	0.04	0.006
Ileum	A	306.66 ± 5.79	72.63 ± 2.01 ^a^	4.22 ± 0.04 ^b^
	B	326.33 ± 7.22	61.69 ± 0.05 ^b^	5.29 ± 0.12 ^a^
	C	284.19 ± 22.67	59.77 ± 1.47 ^b^	4.74 ± 0.27 ^ab^
*p*-value		0.189	0.001	0.013
Duodenum	A	507.31 ± 1.28 ^b^	107.63 ± 4.36	4.73 ± 0.20 ^b^
	B	621.20 ± 9.19 ^a^	110.07 ± 0.81	5.65 ± 0.07 ^a^
	C	608.67 ± 45.32 ^a^	99.41 ± 3.18	6.08 ± 0.28 ^a^
*p*-value		0.046	0.116	0.009

^a, b, c^ Means in the column without common superscripts are significantly different (*p* < 0.05). A, inorganic trace elements; B, 500 mg/kg coated inorganic compound trace elements; C, 1000 mg/kg coated inorganic compound trace elements.

**Figure A1:**
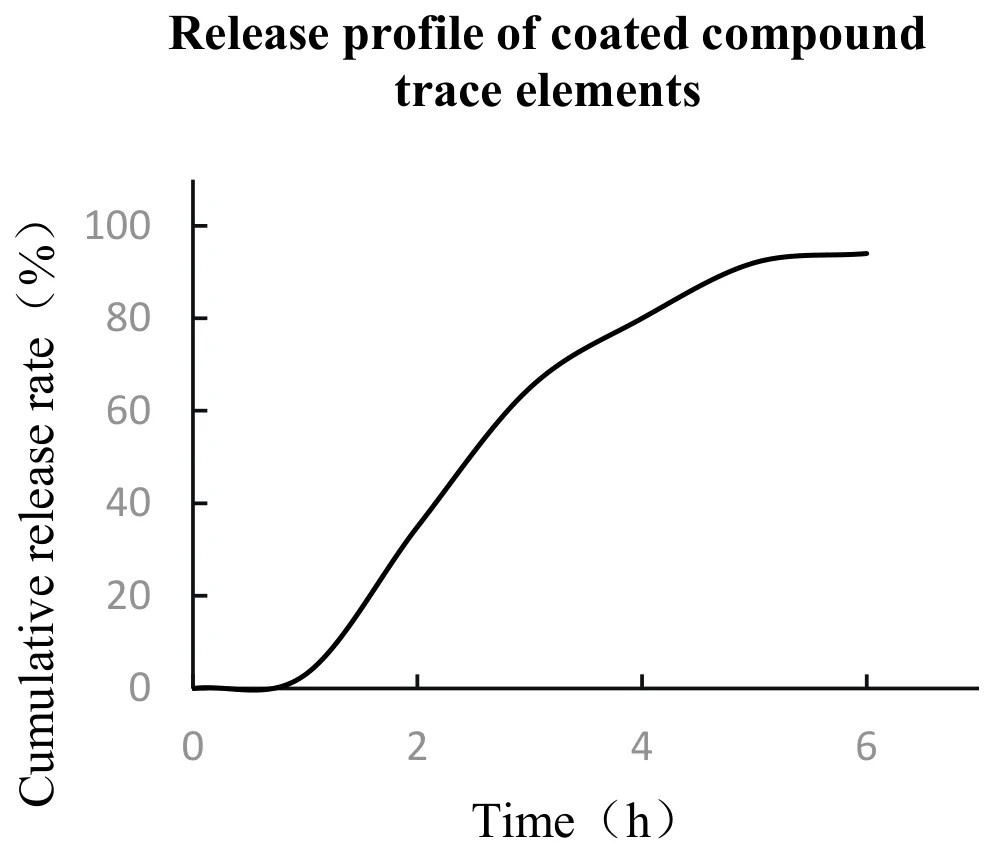
In vitro cumulative release profile of coated compound trace elements.

## Data Availability

The original contributions presented in this study are included in the article/Appendix A. Further inquiries can be directed to the corresponding authors.

## Appendix A

**Table A1:** Estimated daily intake of individual trace minerals per hen (mg/hen/day).

Element ^1^	A	B	C
Cu	2.02	0.65	1.3
Fe	8.58	1.24	2.47
Zn	14.49	5.79	11.55
Mn	14.42	7.21	14.38
Se	0.039	0.024	0.047
I	0.127	0.047	0.094

^1^ A, inorganic trace elements; B, 500 mg/kg coated compound trace elements; C, 1000 mg/kg coated compound trace elements.

**Table A2:** Effects of coated compound trace elements on laying performance of 40-50-week-old egg breeder hens.

Period	Group	Laying Rate (%)	Qualified Egg Rate (%)	Cracked Egg Rate (%)
	A	72.00 ± 2.45	96.75 ± 1.55	0
1	B	75.95 ± 1.83	98.16 ± 0.75	0
	C	75.00 ± 1.17	99.15 ± 0.65	0
	*p*-value	0.334	0.313	-
	A	73.19 ± 1.13 ^b^	94.58 ± 1.50	0.80 ± 0.20
2	B	79.76 ± 1.72 ^a^	97.56 ± 1.01	0.35 ± 0.21
	C	78.57 ± 2.54 ^a^	98.74 ± 0.63	0.36 ± 0.23
	*p*-value	0.033	0.054	0.276
	A	78.69 ± 0.07 ^b^	95.26 ± 1.36	0.74 ± 0.53
3	B	85.00 ± 1.58 ^a^	96.59 ± 0.72	0
	C	85.94 ± 0.94 ^a^	97.20 ± 1.36	0.35 ± 0.21
	*p*-value	0.043	0.517	0.323
	A	80.45 ± 1.37 ^b^	95.86 ± 1.58	0.37 ± 0.22
4	B	86.90 ± 1.43 ^a^	97.81 ± 0.81	0
	C	85.94 ± 1.71 ^a^	98.07 ± 1.10	0.35 ± 0.21
	*p*-value	0.029	0.259	0.300
	A	81.17 ± 3.40	96.16 ± 0.88	0.17 ± 0.17
5	B	84.28 ± 2.17	96.44 ± 1.34	0.16 ± 0.16
	C	86.02 ± 2.60	97.23 ± 0.85	0.18 ± 0.18
	*p*-value	0.478	0.308	0.998
	A	80.58 ± 1.00	96.18 ± 2.24	0.18 ± 0.18
6	B	85.00 ± 2.29	96.19 ± 0.73	0
	C	84.36 ± 2.99	94.99 ± 0.89	0.53 ± 0.22
	*p*-value	0.187	0.801	0.108
	A	79.21 ± 2.00	92.84 ± 1.52	0.92 ± 0.43 ^a^
7	B	85.00 ± 1.93	93.87 ± 0.90	0 ^b^
	C	85.01 ± 2.33	95.47 ± 1.48	0.19 ± 0.19 ^ab^
	*p*-value	0.48	0.4	0.08
	A	82.51 ± 1.67 ^b^	92.98 ± 0.82 ^b^	0.68 ± 0.31
8	B	89.05 ± 1.14 ^a^	95.94 ± 0.56 ^a^	0.36 ± 0.23
	C	86.99 ± 2.15 ^ab^	96.27 ± 0.38 ^a^	0.73 ± 0.53
	*p*-value	0.045	0.005	0.764
	A	79.04 ± 1.60 ^b^	94.18 ± 0.82	0.31 ± 0.31
9	B	81.42 ± 1.35 ^ab^	96.01 ± 0.74	0.23 ± 0.23
	C	84.86 ± 1.23 ^a^	96.21 ± 0.61	0.18 ± 0.18
	*p*-value	0.04	0.129	0.934
	A	78.10 ± 1.54 ^b^	93.66 ± 0.40 ^b^	0.34 ± 0.21
10	B	86.68 ± 2.93 ^a^	94.18 ± 0.07 ^b^	0.17 ± 0.17
	C	86.48 ± 2.04 ^a^	95.47 ± 0.59 ^a^	0.34 ± 0.21
	*p*-value	0.030	0.025	0.784

^a, b^ Means in the column without common superscripts are significant different (*p* < 0.05). A, inorganic trace elements; B, 500 mg/kg coated compound trace elements; C, 1000 mg/kg coated compound trace elements.

**Table A3:** Effects of coated compound trace elements on laying performance of 40–50-week-old egg breeder hens.

Period	Group	Average Egg Weight (g)	Average Daily Feed Intake (g)	Feed to Egg Ratio
	A	60.05 ± 0.70	117.13 ± 1.12	2.51 ± 0.06 ^a^
1~2	B	60.82 ± 0.89	109.38 ± 1.37	2.37 ± 0.07 ^b^
	C	61.64 ± 0.25	116.76 ± 1.06	2.35 ± 0.07 ^b^
	*p*-value	0.281	0.001	0.021
	A	61.05 ± 1.45	121.13 ± 1.99	2.54 ± 0.02 ^a^
3~4	B	61.35 ± 0.60	118.73 ± 0.05	2.28 ± 0.04 ^b^
	C	63.10 ± 1.92	121.18 ± 1.25	2.30 ± 0.05 ^b^
	*p*-value	0.492	0.464	0.047
	A	61.53 ± 0.93	119.86 ± 1.78	2.50 ± 0.07 ^a^
5~6	B	61.82 ± 0.70	118.81 ± 1.50	2.27 ± 0.06 ^b^
	C	62.70 ± 0.13	119.05 ± 0.78	2.25 ± 0.07 ^b^
	*p*-value	0.685	0.474	0.041
	A	61.42 ± 0.47	122.33 ± 1.71	2.47 ± 0.10
7~8	B	61.78 ± 0.89	121.89 ± 1.44	2.39 ± 0.09
	C	62.63 ± 0.21	119.55 ± 1.11	2.31 ± 0.09
	*p*-value	0.366	0.373	0.516
	A	61.25 ± 0.42	122.72 ± 1.76	2.61 ± 0.08 ^a^
9~10	B	62.12 ± 0.79	119.40 ± 1.16	2.26 ± 0.08 ^b^
	C	62.55 ± 0.20	121.34 ± 2.04	2.28 ± 0.06 ^b^
	*p*-value	0.892	0.408	0.036

^a, b^ Means in the column without common superscripts are significant different (*p* < 0.05). A, inorganic trace elements; B, 500 mg/kg coated compound trace elements; C, 1000 mg/kg coated compound trace elements.
